# “It is not just about the alcohol”: service users’ views about individualised and standardised clinical assessment in a therapeutic community for alcohol dependence

**DOI:** 10.1186/s13011-016-0070-5

**Published:** 2016-07-19

**Authors:** Paula Cristina Gomes Alves, Célia Maria Dias Sales, Mark Ashworth

**Affiliations:** Instituto Universitário de Lisboa (ISCTE-IUL), Cis-IUL, Avenida das Forças Armadas, Edifício I, 2w17, 1649-026 Lisboa, Portugal; Centro de Psicologia da Universidade do Porto, Instituto Universitário de Lisboa (ISCTE-IUL), Cis-IUL; Faculdade de Psicologia e de Ciências da Educação, Rua Alfredo Allen, 4200-135 Porto, Portugal; Division of Health and Social Care Research; Faculty of Life Sciences & Medicine, King’s College London, 3rd Floor Addison House, Guy’s Campus, SE1 1UL London, UK

**Keywords:** User involvement, Clinical assessment, Personalised assessment, Evaluation measures, Patient views, Individualised measures, Qualitative research

## Abstract

**Background:**

The involvement of service users in health care provision in general, and specifically in substance use disorder treatment, is of growing importance. This paper explores the views of patients in a therapeutic community for alcohol dependence about clinical assessment, including general aspects about the evaluation process, and the specific characteristics of four measures: two individualised and two standardised.

**Methods:**

A focus group was conducted and data were analysed using a framework synthesis approach.

**Results:**

Service users welcomed the experience of clinical assessment, particularly when conducted by therapists. The duration of the evaluation process was seen as satisfactory and most of its contents were regarded as relevant for their population. Regarding the evaluation measures, patients diverged in their preferences for delivery formats (self-report vs. interview). Service users enjoyed the freedom given by individualised measures to discuss topics of their own choosing. However, they felt that part of the standardised questions were difficult to answer, inadequate (e.g. quantification of health status in 0–20 points) and sensitive (e.g. suicide-related issues), particularly for pre-treatment assessments.

**Conclusions:**

Patients perceived clinical assessment as helpful for their therapeutic journey, including the opportunity to reflect about their problems, either related or unrelated to alcohol use. Our study suggests that patients prefer to have evaluation protocols administered by therapists, and that measures should ideally be flexible in their formats to accommodate for patient preferences and needs during the evaluation.

## Background

Most mental health literature is based on a professional perspective, generated by researchers or practitioners [1]. However, service users have expertise by experience, which is why their involvement is increasingly acknowledged as a crucial part of the health care agenda [2–5].

One area where service user involvement is paramount is the selection of measures to evaluate the patient’s clinical condition [6–8]. Evaluation measures are helpful for clinical work at different points in time during treatment. At treatment intake, they allow the assessment of patients’ distress, and if administered at pre-post treatment, they provide data for outcome assessment purposes. Several authors have suggested that, to maximize their clinical utility, these measures should be relevant, acceptable and valuable for both professionals and service users [6, 9]. The reality, though, is that many popularly used measures do not reflect the service users’ perspective [10–12]. Consequently, we have little information on whether the existing evaluation tools are meaningful, personally relevant, and expressed in terms which make sense to users [6, 8, 13].

User involvement in health care is even more challenging among socially excluded and stigmatised groups, since their views tend to be discredited, undermined and regarded as unworthy [14–17]. This often applies to patients in substance use disorder treatment services, who seldom participate actively in shared decision-making activities [14, 18, 19].

Just as with patients in general, patients in substance use disorder treatment services have firsthand knowledge about their clinical condition and are in a privileged position to inform providers about which outcomes of interest best reflect their reality [14, 20]. According to the European Monitoring Centre for Drugs and Drug Addiction, there are currently over 50 tools to measure treatment outcomes in this population. The vast majority of these are standardised, and do not take the patients’ perspective into account. A recent study gathered 76 variables commonly used by professionals to evaluate recovery from substance use disorder, and service users were asked their views about those criteria [20]. Patients reported that some variables were unrealistic and hard to achieve (e.g. to be completely anxiety-free). This study also highlighted the frustration expressed by patients that most existing variables did not capture individual idiosyncrasies and personal preferences, stating that service providers “had no idea of their experiences” (p. 31).

There has been a recent call for the use of individualised data in the evaluation of substance use disorder treatment [18, 21, 22]. Such data can be collected with individualised measures, which are tailor-made lists of items (problems or goals), generated in patients’ own words [23]. Similarly to pre-set standardised measures, these individualised items are rated for intensity in quantitative scales (e.g. Likert scales). This allows an evaluation of patients’ level of distress, based on their unique problems.

Our study seeks to address three main concerns in this field. First, there are a growing number of studies exploring what users of mental health services think about clinical assessment, including views about the measures and the process by which they are administered [24]. With the exception of the study by Neale and colleagues [20], little is known about what patients in substance use disorder treatment think about clinical assessment. Second, a pioneer study published by Duong et al. [25] has compared patients’ perspectives about standardised and individualised measures in school mental health. To the best of our knowledge, there are no reports on the use of individualised measures in the field of substance use disorder treatment, nor do we know how this population perceives such measures. Third, the literature has suggested that the majority of measures and patient-focused materials in substance use disorder treatment tend to require literacy skills above the average level of literacy among this population [26, 27]. However, those who are most likely to have low reading / writing skills (e.g. low socio-economic status, limited education, marginalised populations, and rural settings) are seldom asked to contribute with their views on clinical assessment.

We were interested in understanding what patients in substance use disorder treatment services, with low literacy skills, think about clinical assessment, in general, and in particular about standardised and individualised measures. More specifically, our aims for this study were two-fold: to explore patients’ overall perspectives about their experience with the evaluation process; and to investigate patients’ views about what is helpful and hindering about each of the four measures in the evaluation protocol. Ultimately, our goal was to understand what makes patients engage, feel (de)motivated or (un)comfortable whilst using evaluation measures as part of their treatment.

## Methods

A single focus group with 10 service users was conducted in a therapeutic inpatient community for females with alcohol dependence, based in a rural area of northern Portugal. This service targets women with severe alcohol dependence problems, who are referred to this facility by local drug and alcohol outpatient units, child protection and social security services and general practitioners. The treatment programme in this facility lasts approximately for 8 months.

On sample characteristics, service users had a mean age of 45 years (SD = 7). Six had completed primary school,^1^ whilst the remaining four were illiterate. The majority were unemployed (six participants) and nearly all (eight participants) had a previous history of substance use treatment episodes. The group took place in the community and was moderated by the first author (PA), assisted by the community’s therapist. Ethical approval was granted by the community’s clinical director. As explained earlier, we opted for a sample with these characteristics (i.e. severe addiction problems, disadvantaged socio-economic status, low literacy skills) since this is likely to represent patients with greater difficulties understanding evaluation measures.

The evaluation protocol used in the therapeutic community consisted of four measures. Two were standardised measures, namely, Treatment Outcomes Profile (TOP; [28]) and Clinical Outcome Routine Evaluation – Outcome Measure (CORE-OM; [29]); and two were individualised, Psychological Outcome Profiles (PSYCHLOPS, [30]) and Personal Questionnaire (PQ; [31]) (see Table 1 for more information). These measures were chosen for being widely used in an international context.

**Table 1:** Summary of measures used in the research protocol

Type of measure	Measure	Generation of items/problems	Domains covered	Nr. items	Size (A4 pages)	Type of items	Delivery format	Example of item
Standardised	TOP	Researchers/professionals	Drug and alcohol use, injecting risk behaviours, offending and criminal involvement, health and social functioning	21	1	Yes/no, Sliding scales	Interview	Q3^a^: “Committed assault or violence”
	CORE-OM	Researchers/professionals	Subjective well-being, symptoms, functioning, risk	34	2	Likert scale	Self-report	Q2: “I have felt tense, nervous or anxious”
Individualised	PSYCHLOPS	Patients	Any of patient’s own choosing	4	1	Likert scale	Self-report	Q1 response: “People in my neighborhood disrespect me”
	PQ	Patients	Any of patient’s own choosing	Unlimited	2	Likert scale	Interview	PQ response: “I miss my family”

^a^“Q”, followed by a number (“Q1”) refers to the number of the item in the questionnaire.

The focus group was conducted in December 2013 and lasted for 2.5-3 h. Eight participants completed the measures at treatment intake only (between Oct-Nov 2013). The remaining two completed the measures twice i.e. at treatment intake (June 2013) and 7 months after (December 2013).

The group discussion was guided by a semi-structured interview focusing on patients’ views about: 1) the evaluation process, i.e. overall satisfaction, duration in time, administration and adequacy of contents of the evaluation protocol; and 2) the helpful and hindering characteristics of each measure in the evaluation protocol i.e. questionnaire length, delivery format and topics covered by the items.

The session was audio-recorded and transcribed verbatim. The transcripts were analysed following a framework synthesis approach [32], based on categories, created a priori, that reflected the information which we aimed to extract (i.e. general aspects about the evaluation process and helpful and hindering aspects of each measure). Data extraction and synthesis was made by one of us (PA) and later discussed with two senior academics (CS and a senior lecturer in Philosophy with expertise in health ethics).

## Results

### General views about the evaluation process

The evaluation process was reported by most service users as a positive experience, because it helped them to reflect about their clinical situation. The overall duration of the evaluation protocol was considered as adequate (“The bigger it is, the more we discover things that we did not know about ourselves”, P7^2^). Patients found it helpful to have their own therapist administering the measures, since “these things are very intimate… if it wasn’t our therapist, we wouldn’t have cared” (P4).

Among those that completed the questionnaires twice, patients felt that certain topics had been difficult to address at treatment intake (“The questions are not wrong, but we’re not used to being honest with ourselves, I was still sort of numb”, P9). However, when answering later in treatment, another patient reported that the questionnaires made her aware of how much she had changed since starting the therapeutic community programme (“It made me think about how different I am. When I arrived I was at the bottom and now I am a new woman”, P7). Patients also considered that all evaluations performed after treatment intake should have been focused on other aspects besides their personal problems, particularly their progress in treatment and the changes that they perceive (“We were given the chance to talk about the problems that we still had, but we could also talk about how we were recovering (…) and I have come such a long way”, P7).

### Helpful and hindering aspects of the evaluation measures

Nearly all measures in the protocol were deemed as adequate in their length, except for CORE-OM, which was considered as “too big” (P4). There was some variability regarding the preferred delivery format, with some patients finding the self-report structure to be more appealing, as “it was easier to tick boxes… we don’t have to think so hard about our problems” (P9) and that “we can be more honest by using a pen” (P3); and others reporting that “if we are forced to talk, it is better because we end up saying something” (P7). Regarding the topics covered by the items, particularly among the standardised measures, there were certain questions that patients found impropriate and hard to answer. Table 2 summarizes the helpful and hindering aspects of each measure as identified by patients.

**Table 2:** Helpful and hindering aspects of TOP, CORE-OM, PSYCHLOPS and PQ as reported by patients

	Helpful		Hindering	
	Key aspects	Patients’ voices	Key aspects	Patients’ voices
TOP	Raises awareness about the quantity of drugs/alcohol used Promotes emotional/breakthrough experiences	“*It is a way of getting yourself together, we have no idea about how much alcohol we used to drink and the money we spent”,* P3	0–20 scale questions to rate psychological/physical health and quality of life difficult to understand and meaningless	*“When I was asked about this I answered by chance. It meant nothing to me. Later we are able to answer in another way”,* P5
CORE-OM	User-friendly Contents relevant to this population Enhances self-awareness	*“This instrument is related to what we are*”, P7	Large number of items Contains questions about sensitive topics (e.g. suicide) Items not generated by patients	*“The questions were made by other people and the words didn’t come from inside of us”*, P7
PSYCHLOPS	Easy to understand Helps reflecting about personal difficulties Provides freedom of expression to talk about any topic, related or not to substance use Makes patients feel like “normal” people	*“It not just about the alcohol, we feel bad about many other things in life. My sister doesn’t drink alcohol but could answer this too, because everyone has problems”,* P8	Requires personal exposure The self-completion format may lead to misleading or incomplete answers	*“We want to hide our real problems for fears of being judged (…) if the words are already written by someone else, it is easier to just say yes or no”,* P7
PQ	Opportunity for self-reflection Oral format encourages to talk about personal problems	*“When a person encourages us to talk, we become more comfortable and open. I talked about my drinking problem...”* P7	Patients reported none.	*“It is fine as it is”,* P1

## Discussion

The purpose of this study was to explore the thoughts of a sample of patients in substance use disorder treatment about the process of clinical assessment. It also aimed to hear those patients’ voices about the characteristics of four evaluation measures that all of them used at treatment intake, and some also later in treatment. Among these were two individualised measures, in addition to two traditional and widely used standardised measures.

Our first goal was to investigate patients’ general views about the evaluation process and the findings were encouraging. We learned that patients not only welcomed clinical assessment, but also perceived it as a valuable task for their therapeutic journey. Patients were satisfied with the duration of the evaluation protocol (which included six A4 size pages) and there was even openness for the inclusion of further items. Previous studies [6, 7] have shown that patients tend to be concerned about the brevity of several measures, for being “too simplistic”. In contrast, studies of services and therapists, report that evaluation measures can become a burden for patients and potentially interfere with the time assigned for the consultations and treatment [33].

There was a general preference to have therapists administering the evaluation protocol, making it a meaningful part of the therapeutic process and potentially leading to a greater commitment with the task. As such, we believe that clinical assessment could be formally included as part of treatment, which has already been proposed by authors such as Valderas [34]. The major advantage of this is that using evaluation measures would not require extra human and time resources from the service, making it a potentially more feasible task in real clinical settings. As a downside, one must bear in mind that when therapists administer the protocol directly, patients’ answers are likely to be biased, particularly in oral interviews. In such cases, patients may feel the need to provide desirable answers and underreport undesirable behaviours, to satisfy their therapist, as reported by Bowling [35]. However, unless patients are under court-ordered treatment, they tend to be disposed and motivated to disclose personal and clinically relevant information to their therapists. Hence, we believe that if the interviewer is also the therapist, the risk of offering socially desirable answers is likely to decrease. Considering that most research about social desirability in mental health has been conducted with non-clinical samples [35] further studies are needed to ascertain the pros and cons of having therapists as interviewers in clinical assessment, which is something that, as we have seen, patients seem to prefer.

Our second main goal was to learn what was helpful and hindering about the measures in the evaluation protocol, from the patient perspective. There was a tension regarding service users’ preferences about the delivery format of measures, with some favouring the simplicity of ticking boxes, and others keener on talking about their problems. This suggests that a one-size-fits-all approach to evaluation is not enough and flexibility is desirable, so that patients’ preferences can be considered. Such flexibility has already been suggested by Gordon and colleagues [24]. As such, we need to further explore to what extent the psychometric properties of an instrument remain unaltered in multiple formats of application, i.e. allowing a flexible administration of measures while providing reliable information for treatment evaluation.

However, when it came to eliciting personalised information, most patients in our group preferred the dialogue, oral format of PQ, rather than describing their problems in writing, as required by the PSYCHLOPS questionnaire. This is consistent with the study by Ashworth and colleagues [36], where therapists felt that PSYCHLOPS was challenging because patients not only had to identify problems on their own, but also to use their own words to write their problems down.

In our study, standardised and individualised measures were seen as relevant for clinical assessment, despite having certain disadvantages. TOP and CORE-OM were perceived as useful and relevant for this population, suggesting a good level of acceptability among patients. Nevertheless, not all contents covered by these two standardised measures were regarded as meaningful or appropriate (e.g. rating psychological health in a 20-point scale). Also, service users expressed some reservations about the disclosure of sensitive personal information in certain TOP and CORE-OM items, as shown in other studies [37]. One likely consequence of patients feeling uncomfortable or dissatisfied with the evaluation questionnaires is the likelihood of misleading and/or missing responses. Thus, further research is needed to ascertain which topics are likely to trigger negative reactions to the evaluation process.

As expected, patients appreciated the freedom given by both individualised measures, PSYCHLOPS and PQ, to express any type of personal concern, regardless of topic. This was in line with Duong and colleagues [25], who demonstrated that recipients of mental health care consider individualised measures to be less confining than their standardised counterparts. Hence, our findings indicate that accommodating a great diversity of topics is important to patients, since misusing substances can lead to/or be the consequence of problems that drug-focused instruments might not address. Future research should compare the topics elicited from standardised and individualised measures, so that we understand if the former tend to overlook aspects of relevance for patients that the latter are able to capture.

Finally, it is also worth emphasizing that patients who responded to the evaluation measures at treatment intake and later in treatment valued the opportunity to focus on other aspects besides outcomes. This could be overcome by including items about the treatment process, giving patients the opportunity to share their thoughts about the care they are receiving. Such feedback about treatment could be used by clinicians to adjust the intervention to match the patient’s needs, as well as to increase therapeutic alliance [38].

This study is not without limitations. To have a female only, small sample size means that the findings are less generalisable and conclusions should be interpreted with caution. Also, the presence of the patients’ therapist in the group may have overstated their positive views about the evaluation process and the measures included in our study.

## Conclusions

This study suggests that service users can actively contribute to improving the process of clinical assessment, guiding researchers and professionals towards developing evaluation measures that are more meaningful and relevant for patients with alcohol dependency. Individualised outcome measures have the potential to broaden the range of viewpoints captured from patients compared to the more narrowly focused standardised instruments.

## Abbreviations

CORE-OM, clinical outcome routine evaluation – outcome measure; PQ, personal questionnaire; PSYCHLOPS, psychological outcome profiles; TOP, treatment outcomes profile

## Footnotes

1 In Portugal, the term “primary school” refers to 4 years of education, from the age of 6 to 10, and is also known as the 1^st^ cycle of basic education.

2 “P”, followed by a number (e.g. “P3”) is an anonymous designator for each focus group participant.
